# Studying the Effect of High Substrate Temperature on the Microstructure of Vacuum Evaporated TAPC: C_60_ Organic Solar Thin Films

**DOI:** 10.3390/ma14071733

**Published:** 2021-04-01

**Authors:** Mohamed Abdelaal, Mohamed Hazem Abdellatif, Moritz Riede, Ghada Bassioni

**Affiliations:** 1 Faculty of Engineering, Ain Shams University, Cairo P.O. Box 11517, Egypt; mohamed.abdelaal@physics.ox.ac.uk (M.A.); mohamed_abdellatif@eng.asu.edu.eg (M.H.A.); 2 Department of Physics, University of Oxford, Oxford OX1 3PU, UK; moritz.riede@physics.ox.ac.uk

**Keywords:** renewable energy, organic semiconductors, organic solar cells, vacuum evaporation, X-ray diffraction, microstructure

## Abstract

Organic solar cells (OSCs), also known as organic photovoltaics (OPVs), are an emerging solar cell technology composed of carbon-based, organic molecules, which convert energy from the sun into electricity. Key for their performance is the microstructure of the light-absorbing organic bulk heterojunction. To study this, organic solar films composed of both fullerene C_60_ as electron acceptor and different mole percentages of di-[4-(N,N-di-p-tolyl-amino)-phenyl]-cyclohexane (TAPC) as electron donor were evaporated in vacuum in different mixing ratios (5, 50 and 95 mol%) on an ITO-coated glass substrate held at room temperature and at 110 °C. The microstructure of the C_60_: TAPC heterojunction was studied by grazing incidence wide angle X-ray scattering to understand the effect of substrate heating. By increasing the substrate temperature from ambient to 110 °C, it was found that no significant change was observed in the crystal size for the C_60_: TAPC concentrations investigated in this study. In addition to the variation done in the substrate temperature, the variation of the mole percent of the donor (TAPC) was studied to conclude the effect of both the substrate temperature and the donor concentration on the microstructure of the OSC films. Bragg peaks were attributed to C_60_ in the pure C_60_ sample and in the blend with low donor mole percentage (5%), but the C_60_ peaks became nondiscernible when the donor mole percentage was increased to 50% and above, showing that TAPC interrupted the formation of C_60_ crystals.

## 1. Introduction

Burning fossil fuels changes the radiation balance and leads to climate change by emitting large amounts of greenhouse gases [1]. Fossil fuels are neither sustainable, given their limited amounts, nor environmentally friendly. We therefore need to find alternatives. Solar energy is one of these alternatives and the only one that can in principle power all of society.

Organic solar cells are made from organic, i.e., carbon-based molecules, similar to the ones found in organic light-emitting diode (OLED)-based displays of many mobile phones, whereas conventional solar cells are made from inorganic materials, most commonly silicon. Inorganic solar cells (ISCs) are opaque and not mechanically flexible in general [2]. However, commercial ISCs currently have better power conversion efficiency (PCE) and longer lifetime compared to OSCs, and ISCs are dominating the market [3,4].

Organic solar cells have great potential for providing inexpensive and more flexible energy options, in addition to the fact that they can be made semi-transparent [5]. Scientists are therefore working to boost the performance of OSCs and improve their efficiency and lifetime. The thin film microstructure of the active layer can affect both and hence, it is important to investigate and understand the effect that the microstructure has. The microstructure of the evaporated thin-films for OSC is usually amorphous or polycrystalline with up to several crystalline polymorph components. Numerous factors, such as crystalline orientation, domain size and purity influence the optical and electronic properties [6,7,8,9,10,11,12,13,14].

One way of tuning the microstructure of vacuum deposited films, i.e., the technology that is dominating the commercial production of OLEDs, is to control the substrate temperature T_sub_ during the deposition. By increasing T_sub_, the molecules are more mobile due to a more energetic environment on the surface, which can lead to the formation of larger domains and crystals. In this paper we study this effect by X-ray scattering for different concentrations of TAPC (di-[4-(N,N-di-p-tolyl-amino)-phenyl]-cyclohexane) and fullerene C_60_, two model compounds used for OSC, which were deposited at different T_sub_. Grazing incidence X-ray scattering (GIXS) provides valuable unique insights into the nature of the active materials used in organic photovoltaic devices and thin film layers. Grazing incidence wide angle X-ray scattering (GIWAXS) geometry enables the determination of the crystal structure and the orientation of the crystalline regions [15].

The influence of raising the substrate temperature from room temperature to 110 °C was studied previously on ZnPc: C_60_ blends and revealed an increase by 1% absolute in the efficiency of the OSC, which was due to an increase in the photocurrent and fill factor (FF). This was correlated to a more favorable microstructure in the blend, which in turn led to a better charge carrier extraction [16]. In addition to that, another study investigated the effect of thermal annealing at 100, 200, 300 and 400 °C temperatures for C_60_ films evaporated on silicon surface at low temperatures, and the results indicated that the C_60_ films annealed at 100 °C have the highest packing density, low surface roughness, high degree of crystallinity and stable ohmic contacts [17]. In our research, we investigated the effect of high substrate temperature (110 °C) during deposition on the C_60_: TAPC films evaporated on ITO glass for the different C_60_: TAPC concentrations.

High-efficiency organic solar cells are most commonly based on a bulk heterojunction (BHJ) structure, consisting of a thin film of mixed electron donor and acceptor molecules. To achieve high-power efficiency, the donor: acceptor concentration needs to be adjusted for optimum light absorption and charge carrier extraction. It was shown that a large open-circuit voltage (V_oc_ > 1.0 V) can be achieved in fullerene-based OSC with almost any donor, provided that the donor is in low concentration. The best photovoltaic performance was obtained in cells with a low TAPC concentration of about 5–10 mol% compared to high TAPC concentration of up to 50 mol% [18].

It was shown in another study that intermolecular charge-transfer (CT) excitons (an exciton is a bound state of an electron and a hole, which are bound to each other by the electrostatic coulomb force) in the C_60_: TAPC dilute BHJs rapidly localize to Frenkel excitons prior to dissociating at the donor–acceptor interface. Thus, the entire Frenkel and charge-transfer range of the fullerene absorption can thus be exploited for charge generation, which makes the C_60_: TAPC an ideal system to be studied since the charge-transfer state energy does not change with mixing ratios [19].

Based on the outcomes of the above studies showing the potential of the dilute C_60_: TAPC as BHJ blends, in our research, the low donor concentration (5 mol% TAPC) was investigated as the optimum donor concentration and was compared with high donor concentration of 50 and 95 mol% and with the pure C_60_ sample with variant substrate temperatures.

**Figure 1:**
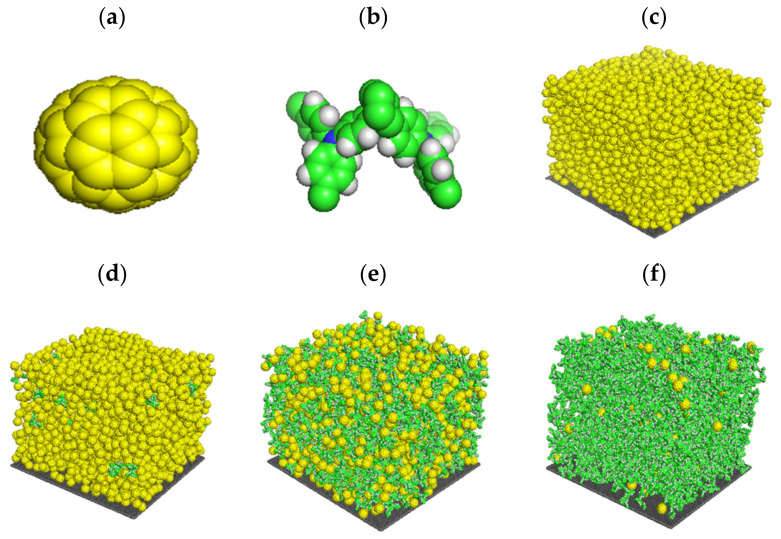
C_60_ is shown in (**a**) and the di-[4-(N,N-di-p-tolyl-amino)-phenyl]-cyclohexane (TAPC) is shown in (**b**). (**c**) Shows the film structure for the pure C_60_ sample, (**d**) shows the film structure after adding 5 mol% of TAPC. (**e**) Shows C_60_ with 50 mol% TAPC and (**f**) shows the film structure of C_60_ with 95 mol% TAPC. All these film structures were drawn using GROMACS (Reprinted from ref. [21].) based on GROMOS 54A7 forcefield and visualized by Pymol.

Another study initially investigated the effect of TAPC on the microstructure of the C_60_ through simulations of the atomistic nonequilibrium molecular dynamics using the GROMACS program for simulations [20]. The simulations were used to model the microstructure of the vacuum-deposited small-molecule bulk heterojunction films as used in organic photovoltaics. Films consisting of C_60_ and 1, 5, 10 and 50 mol% TAPC were compared with films of neat C_60_. Figure 1 shows how the neat C_60_ and the different mole percentages of TAPC (5 and 50%) appear in the simulation. The study showed that by increasing the TAPC content, the roughness, porosity and crystallinity of the films were decreased. This hypothesis was studied here to check if the amount of TAPC affected the crystallinity of the C_60_ as shown by the modelling software [20].

It is also possible that the material’s porosity would affect exciton and charge mobility inside the bulk heterojunction layer [20]. TAPC’s tendency to minimize film roughness, porosity and crystallinity theoretically provides an additional aspect to consider when designing these blends. Knowledge of the microscopic structure of a bulk heterojunction is crucial to obtaining a detailed picture of the local environment in which both the donor and the acceptor are located, such that the optoelectronic properties of such materials can be predicted more accurately [20].

Figure 1 shows the 3D structure of the acceptor (C_60_) colored in yellow and the donor (TAPC) colored in green and white in Figure 1a,b, respectively. Followed by the film structure of the C_60_: TAPC devices investigated in our study. Figure 1c shows the pure C_60_ sample, and Figure 1d shows the C_60_ with 5 mol% of the donor (TAPC). Figure 1e,f shows the stack structure with 50 and 95 mol% TAPC, respectively.

## 2. Materials and Methods

The OSC layers were evaporated in a custom-made vacuum evaporation chamber (CreaPhys GmbH, Dresden, Germany). The substrates used were ITO on glass with sheet resistance equal to 20 ± 2 ohms/sq (TDF Inc, Anaheim, CA, USA) and were first cleaned for 10 min in 2.5% Hellmanex solution water and then for 10 min in DI water followed by 10 min cleaning in acetone, and finally for 10 min in IPA. Subsequently, the substrates were treated with UV-ozone for 10 min prior to loading the substrates into the N_2_-filled glovebox. The organic materials and MoO_x_ were loaded as powder into crucibles in the evaporation chamber. Substrates were subsequently loaded into the chamber from the glove box to avoid air exposure. As a first layer, MoO_x_ (3 nm) was deposited at 0.08 A/s as a hole interface layer to ensure that the subsequent layers grow on the same underlayer as full OSC.

**Figure 2:**
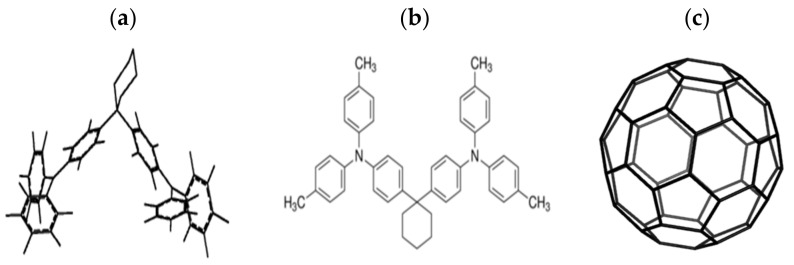
Structures of di-[4-(N,N-di-p-tolyl-amino)-phenyl]- cyclohexane (TAPC) in 3D in (**a**) and in 2D in (**b**) and the C_60_ molecule in (**c**).

Figure 2 shows the structures of the molecules being studied, with the C_60_ molecule having a spherical shape as shown in Figure 2c; TAPC is shown in 3D in Figure 2a and in 2D in Figure 2b, showing the flexible nature of TAPC and likely conformation in the film.

The photovoltaic active layer (50 nm) of C_60_ Fullerene and (5, 50 and 95 mol%) TAPC were co-deposited with the rates mentioned in Table 1. The base pressure of the chamber was kept at 10^−6^–10^−7^ mbar. For all the samples deposited at a high substrate temperature, the substrate was heated, while evaporating the MoO_x_ and the active layer (C_60_: TAPC) for the whole time, and the temperature was transmitted using a copper block, which was connected to the substrate. The temperature was kept constant and was monitored using a thermocouple. The evaporation rate for each molecule varied depending on the TAPC % in the blend, and the overall evaporation rate for the blend was held at approximately 0.4 A/s. Table 1 summarizes the deposition rates used in our study for the different C_60_: TAPC concentrations.

After achieving the target thickness of 50 nm, the substrate deposition chamber was vented to ambient pressure with N_2_ and the samples removed into the attached glovebox where the samples were stored until further investigation including the X-ray diffraction.

The microstructures of the OSCs were examined using X-ray diffraction, Beamline I07 beamtime number NT26630-1 at Diamond Light Source (DLS, Oxford, UK). The 20 keV beam source was calibrated using silver behenate (AgBe) as a reference sample. The setup used a Pilatus P2M detector mounted at a distance of 421 mm for grazing incidence wide angle X-ray scattering (GIWAXS) from the sample to give a potential angular collection range of up to 40°. The GIWAXS spectra including the peak fitting were processed and analyzed using DAWN Science (version2.20.0, Diamond Light Source, DLS, Oxford, UK,) [22,23].

## 3. Results

**Figure 3:**
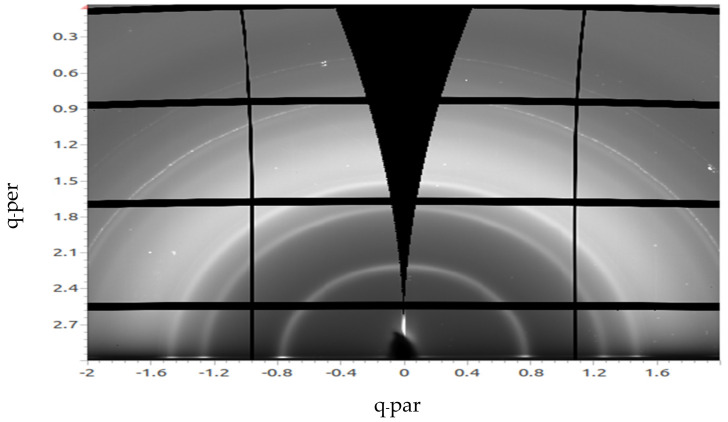
Grazing incidence wide angle X-ray scattering (GIWAXS) image of a 50 nm C_60_ film deposited by vacuum thermal evaporation on top of 3 nm MoOx on a substrate at room temperature.

Figure 3 is a GIWAXS image of a 50 nm C_60_ film deposited by vacuum thermal evaporation on top of 3 nm MoO_x_ evaporated on a substrate held at room temperature. The x-ray peaks appear as semicircle arcs indicating the amount of crystallinity in the C_60_ lattice. Peak fitting using a Gaussian model was performed for all the samples using DAWN. For the pure C_60_, the peak was fitted at a q_z_ value of 0.75 Å^−1^. The Scherrer equation can be used to estimate the crystal size based on the peak position and the FWHM (full width at half maximum) values [24]. The Scherrer equation can be written as:

$$\tau =\;\frac{K\;\lambda }{\beta \;\cos\theta }$$

where *τ* is the average crystallite size in nm,

*λ* is the X-ray wavelength,
*K* is the shape factor,
β is the FWHM of XRD peak and
*θ* is the Bragg angle.

The Scherrer equation gives an estimate of the lower bound of grain size and can be used for estimations of crystal sizes up to 100–200 nm. The crystallite shape was assumed spherical and hence the value of the shape factor used in the calculation was 0.94. Using the Scherrer equation, this peak value belongs to the pure C_60_ sample evaporated at RT and leads to an estimated crystallite size of 10.1 nm. This value is consistent with data mentioned in a previous study [25], in which the investigators obtained a C_60_ crystallite size of 10 nm.

**Figure 4:**
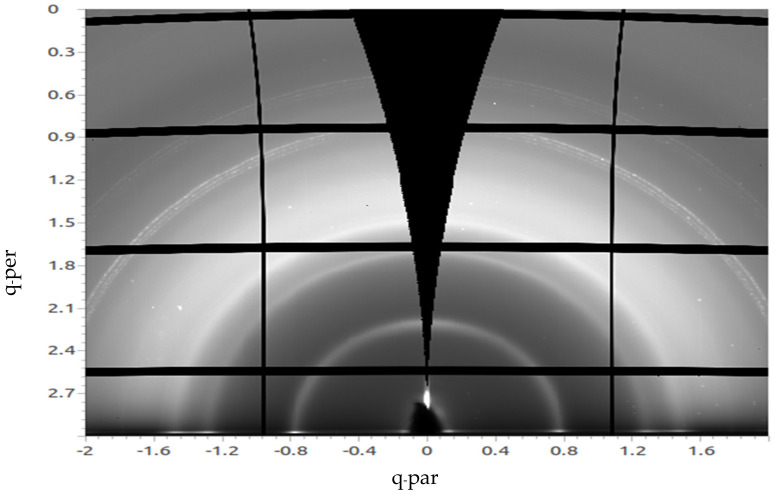
GIWAXS image of a 50 nm C_60_ film deposited by vacuum thermal evaporation on top of 3 nm MoOx at a T_sub_ of 110 °C.

In Figure 4, the GIWAXS image of a 50 nm C_60_ film deposited by vacuum thermal evaporation on top of 3 nm MoO_x_ at a substrate temperature of 110 °C is shown. The semicircle arcs can be seen again. The peak was fitted at a q_z_ value of 0.76 Å^−1^**.** This estimate puts the crystal size to 9.8 nm. The peaks can be also seen clearly and are almost the same as in Figure 3. The fitted peaks for both the C_60_ evaporated at RT and the C_60_ evaporated at 110 °C are almost at the same q_z_ values, hence indicating that the crystal size did not vary significantly and both crystals domain sizes are nearly equivalent.

**Figure 5:**
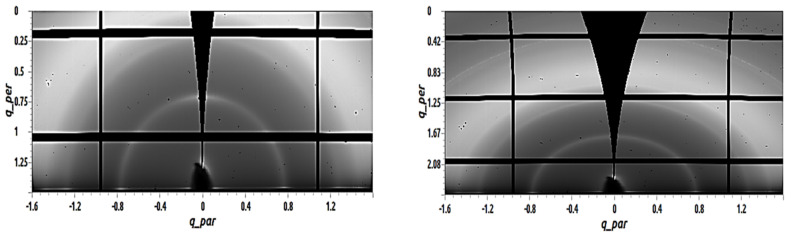
GIWAXS images of 50 nm C_60_ films with 5 mol% TAPC deposited by vacuum thermal evaporation on top of 3 nm MoOx; the sample shown on the left was evaporated on a substrate at RT and the sample shown on the right was evaporated on a T_sub_ = 110 °C.

In Figure 5, with the addition of 5 mol% of TAPC, the semicircle arcs can still be noticed for both, the sample evaporated at RT (left) and the sample evaporated at temperature 110 °C (right), and peaks were again fitted using a Gaussian model. For the C_60_ sample with 5 mol% TAPC evaporated on a substrate at RT shown on the right of Figure 5, the peak was fitted at a q_z_ value of 0.75 Å^−1^. This was translated using the Scherrer equation into an estimated crystal size of 10.44 nm. While for the sample on the right of Figure 5, representing the C_60_ sample with 5 mol% TAPC evaporated on a substrate held at 110 °C, the peak fitting has shown a peak at the q_z_ value of 0.76 Å^−1^ and indicated an estimated crystal size of 9.55 nm. It can be again noted that the C_60_ samples with 5 mol% TAPC evaporated at both RT and 110 °C have comparable calculated grain sizes in the range of 10 nm, similar to pristine C_60_ layers. This indicates that the addition of the 5 mol% TAPC did not have a significant change on the degree of crystallinity and showed no change in the grain size.

**Figure 6:**
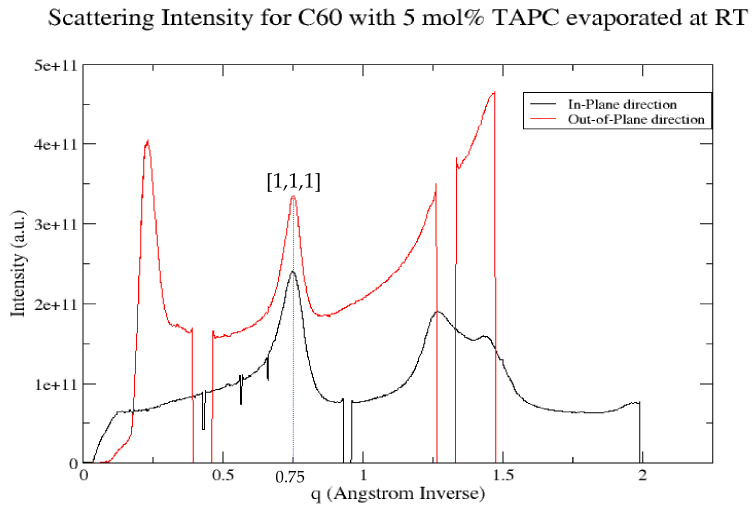
Scattering intensity for the C_60_ film with 5 mol% TAPC evaporated at RT shown in both the in-plane direction taken by a radial slice from the origin at angles 0° to 1° shown in black and the out-of-plane direction taken by a radial slice from the origin at angles of 86° to 87° shown in red.

We noticed an anisotropy of the ring intensity between the in-plane direction taken by a radial slice from the origin at angles 0° to 1° and the out-of-plane direction taken by a radial slice from the origin at angles 86° to 87° for the C_60_ film with 5 mol% TAPC evaporated at RT shown in Figure 5 (left). This was further investigated by drawing the scattering intensity for both the in-plane direction and the out-of-plane direction for the C_60_ film with 5 mol% TAPC evaporated at RT. As shown in Figure 6, there is a difference in the intensity between the in-plane direction and the out-of-plane direction for the [1, 1, 1] peak fitted at the q_z_ value of 0.75 Å^−1^, which is in good agreement with the values obtained in previous studies [26]. As the C_60_ molecule is isotropic, this is difficult to explain. We believe it is due to enhanced scattering due to the Yoneda band for the in-plane direction.

**Figure 7:**
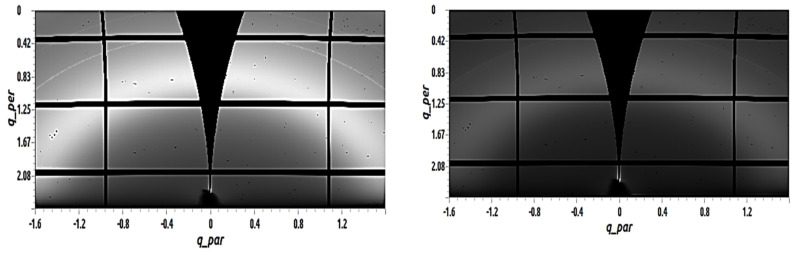
GIWAXS images of 50 nm C_60_ films with 50 mol% TAPC deposited by vacuum thermal evaporation on top of 3 nm MoO_x_. The sample shown on the left was evaporated at RT, and the sample shown on the right was evaporated at T_sub_ = 110 °C.

Figure 7 shows GIWAXS images of 50 nm C_60_ films with 50 mol% TAPC deposited by vacuum thermal evaporation on top of 3 nm MoOx, both at RT (on the left in Figure 6) and at 110 °C (on the right in Figure 6). The semicircle arcs shown previously in the pure C_60_ samples and in the C_60_ samples with 5 mol% TAPC cannot be noticed anymore. As a result, fitting these peaks was not possible. The absence of peaks indicates that by adding 50 mol% of the amorphous TAPC to the C_60_, the crystalline order of C_60_ was disturbed and no peak was able to be fitted for both the sample evaporated at RT and the sample evaporated at T_sub_ = 110 °C.

**Figure 8:**
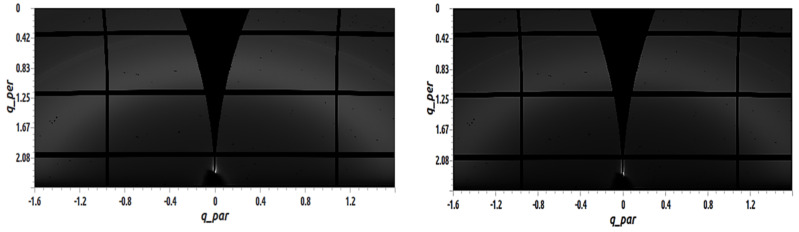
GIWAXS images of 50 nm C_60_ films with 95 mol% TAPC deposited by vacuum thermal evaporation on top of 3 nm MoO_x_. The sample shown on the left was evaporated at RT and the sample shown on the right was evaporated at T_sub_ = 110°C.

Figure 8 shows GIWAXS images of 50 nm C_60_ films with 95 mol% TAPC deposited by vacuum thermal evaporation on top of 3 nm MoO_x_. The sample shown on the left side was evaporated at RT, and the sample shown on the right side was evaporated at 110°C. Since all the semicircle arcs have disappeared in Figure 7, it was not possible to fit any peaks in this blend. The addition of 95 mol% TAPC to the C_60_, as was expected, further reduced the crystallinity of the C_60_.

Table 2 summarizes the peak positions in both q_z_ and the equivalent grain size for the pure C_60_ samples and the C_60_ with 5 mol% TAPC at both RT and at 110 °C. As shown in the Table, the estimated grain size for all the 4 samples is roughly the same with negligible variations with values varying from 9.5 nm, which belongs to the C_60_ sample with 5 mol% TAPC evaporated at 110 °C, up to 10.44, which belongs to the C_60_ sample with 5 mol% TAPC evaporated at RT.

**Figure 9:**
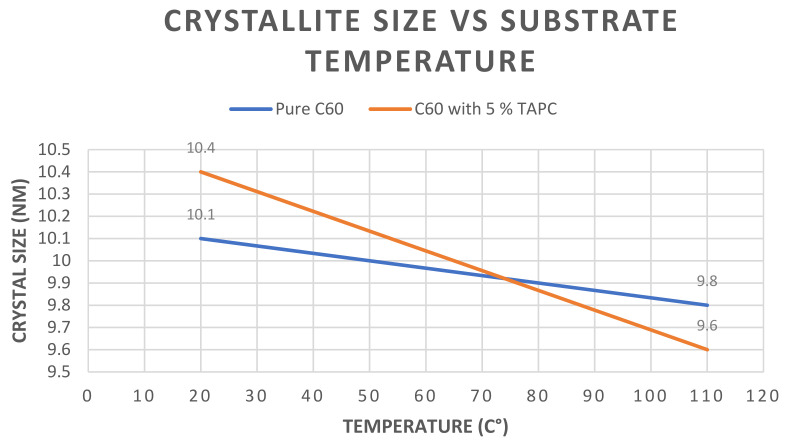
Graphical representation showing the variations in crystallite size (nm) for C_60_ samples and C_60_ with 5% TAPC samples evaporated at both RT (20 °C) and 110 °C.

Figure 9 shows the variations in crystallite size in nm between the pure C_60_ samples in blue and the C_60_ with 5 mol% TAPC samples in red when evaporated at both RT and 110 °C. It is noticed that by increasing the substrate’s temperature from RT to 110 °C, the crystallite size decreased from 10.4 nm to 9.6 nm for the pure C_60_ sample and from 10.1 nm to 9.8 nm for the C_60_ with 5 mol% TAPC sample.

## 4. Discussion

It was possible to fit peaks in the GIWAXS pattern of thin films using a Gaussian model for both the pure C_60_ and the C_60_ with 5 mol% TAPC evaporated at both RT and at 110 °C. This indicates that neither the 5 mol% TAPC nor the evaporation at high substrate temperature (110 °C) disturbed or increased the crystalline order of the C_60_. This was clear as peaks were fitted at almost the same positions showing that roughly the grain sizes were equal to the ones found in pure C_60_ samples. The grain sizes, estimated with the Scherrer formula, were in the same range (9.5–10.5 nm).

By increasing the mol% of the amorphous TAPC to 50 mol% and above to the C_60_ matrix, TAPC disturbed the crystalline order of C_60_ and no peak in the GIWAXS data was able to be fitted for both the sample evaporated at RT and the sample evaporated at T_sub =_ 110 °C. Hence, it was deduced that the substrate temperature has no significant effect on the crystallinity of the C_60_ for the studied blends C_60_: TAPC with the above investigated concentrations. More samples with other TAPC concentration between (5–50% mole) shall be studied to have the complete data on the effect of TAPC on C_60_.

The hypothesis claiming that increasing the T_sub_ to 110 °C would increase the C_60_ crystallinity was not found to apply in this case and did not affect the crystallinity of the C_60_ as no change in the peak sharpness was noticed. Cold, i.e., sub-zero substrate temperatures should be studied to determine the effect of various substrate temperatures varying from cold (negative values) to hot substrate temperature (>110 °C) as studied in this paper.

## Figures and Tables

**Table 1:** The C_60_: TAPC deposition rates for the different concentrations evaporated in our study.

Sample	Deposition Rate of C_60_ in A/s	Deposition Rate of TAPC in A/s
Pure C_60_	0.4	0.0
5% TAPC	0.37	0.03
50% TAPC	0.23	0.17

**Table 2:** The GIWAXS data for the pure C_60_ samples and the C_60_ with 5 mol% TAPC at both RT and 110 °C. The peak positions and the grain size are listed for each of the 4 samples.

Sample	q_z_ Peak Position in Å^−1^	Crystal Size (nm)
C_60_ at RT	0.75	10.1
C_60_ at 110 °C	0.76	9.8
C_60_ with 5 mol% TAPC at RT	0.75	10.4
C_60_ with 5 mol% TAPC at 110 °C	0.76	9.6

## Data Availability

The data that support the findings of this study are available from the corresponding author upon reasonable request.
